# Validating the use of the Wijma Delivery Expectancy/Experience Questionnaire in Mainland China: a descriptive, cross-sectional study

**DOI:** 10.1186/s12884-022-05283-w

**Published:** 2022-12-12

**Authors:** Lu Han, Jiaxin Wu, Hengchang Wu, Jun Liu, Yanqun Liu, Zhijie Zou, Juan Liu, Jinbing Bai

**Affiliations:** 1 School of Nursing, Wuhan University, Hubei, China; 2 School of Public Health, Wuhan University, Hubei, China; 3 Department of Obstetrics and Gynecology, Zhongnan Hospital affiliated with Wuhan University, Hubei, China; 4 Nell Hodgson Woodruff School of Nursing, Emory University, Atlanta, GA USA

**Keywords:** Fear of childbirth, W-DEQ-A, Pregnancy, Childbirth, Psychometric testing

## Abstract

**Background:**

Fear of childbirth (FOC) is a common psychological problem in Chinese pregnant women. FOC can influence both maternal health and infants’ wellness. Special assessment tools for FOC in Mandarin Chinese are currently lacking. The aim of this study was to evaluate the psychometric properties of the Mandarin Chinese of the Wijma Delivery Expectancy/Experience Questionnaire Version A (W-DEQ-A).

**Methods:**

We recruited 364 Chinese pregnant women from April 2021 to July 2021. Translation and cultural adaptation, as well as reliability and validity testing were conducted. Analyses included the content validity, structural validity, criterion-related validity, convergent validity and reliability. The content validity indices were used to assess the content validity of the tool. The structural validity was tested through exploratory factor analysis and confirmatory factor analysis. The Cronbach’s alpha coefficient was used to evaluate the reliability of the W-DEQ-A Chinese version.

**Results:**

The Chinese translation showed excellent similarities and equivalence to the original version, with the satisfactory content validity. Factor analysis indicated 5 factors, accounting for 57% of the total variance. Both criterion-related validity and convergent validity proved to be acceptable. The reliability was tested with a Cronbach’s alpha coefficient of 0.911 for the total scale.

**Conclusion:**

The W-DEQ-A Chinese version is a reliable and valid tool to identify FOC in Mandarin Chinese-speaking populations.

## Background

Fear of childbirth (FOC) is a negative cognitive evaluation, caused by women’s feelings of uncertainty and anxiousness. It is a common psychological problem for women before childbirth [1]. Influencing factors to the extent of FOC have been reported to encompass demographical dimensions (e.g., age, parity [2], educational level [3], etc.), psychological emotions such as fear and trait anxiety [4], lack of disbelief and control about the body’s ability to deliver safely [5], and baby’s safety condition [6]. Furthermore, evidence has shown that women with elevated fears suffer from the negative appraisal of childbirth [7].

FOC can result in a series of adverse consequences. For example, pregnant women doubt their ability to deliver [8] can increase risk of postpartum hemorrhage and suffer from psychological disorders such as depressive disorder and post-traumatic stress disorder [9]. In addition, infants of mothers who experienced FOC are at an increased risk of fetal distress, low Apgar scores, and premature delivery [10], which can further affect children’s learning ability, development, and behaviors. Therefore, FOC is an acute health issue that requires timely identification and appropriate strategies. FOC can also cause an increase in cesarean section without indications, and nulliparous women had a higher incidence of FOC than parous women [11]. Finally, the prevalence of FOC differs from country to country because of the geographical, cultural, and medical differences. Of women in European countries, 11% of women had severe FOC [12]. In China, according to population data from the National Bureau of Statistics of China, the average annual number of births was 16.2 million over the past decade. Remarkably, the total prevalence of FOC was up to 67% [13].

Self-reported instruments have been widely used to assess an individual’s levels of FOC, including Childbirth Attitude Questionnaire (CAQ) [14], Visual Analogue Scale (VAS) [15], Fear of Birth Scale (FOBS) [16], and Wijma Delivery Expectancy/Experience Questionnaire (W-DEQ) [1]. Though the first three scales have good reliability and validity, there are still some notable limitations. For example, the appropriate cut-off score of CAQ has not been confirmed. Additionally, the VAS is not a specific tool for pregnant women so it is only suitable for initial screenings and the FOBS scale usually produces higher FOC rates than real situations [17].

W-DEQ is commonly used to more accurately and effectively measure FOC [18]. This scale is a standardized screening method for FOC and includes two versions, version A and version B, to assess fear about childbirth during pregnancy and after childbirth separately. The aim of the Wijma Delivery Expectancy/Experience Questionnaire version A (W-DEQ-A), which contains 33 items, is to elicit pregnant women’s expectations of birth and can be used extensively to evaluate levels of fear before delivery [1]. It has been proven to have high reliability and validity [19]. Previous research conducted a structural validity analysis of the W-DEQ-A, and the number of dimensions was not uniform across countries [20]. In recent years, the original version of W-DEQ-A has been translated into different languages, such as Danish [21], Turkish [19], Persian [22, 23], and Spanish [18] to name a few.

However, Chinese maternal health is currently more focused on screening and diagnosis of prenatal diseases, and it has paid little attention to FOC. While there is a Cantonese version of W-DEQ-A [24], the lack of a Mandarin (Simplified) Chinese version of W-DEQ-A, limits its potential application to Mandarin Chinese-speaking women. Mandarin (Simplified) Chinese is one of the most common languages, used by nearly one-sixth of the global population. As there are significant differences between the Mandarin (Simplified) Chinese and the Cantonese lexical tones, text features, and grammatical structures, it is imperative that we provide the translated assessment tools to study FOC in the greater population. Hence, the aim of the study was to analyze psychometric properties of the Chinese version of W-DEQ-A, which can probe into its applicability in the Chinese population.

## Methods

### Design, setting, and participants

This was a cross-sectional study. This study used convenience sampling, which was conducted in the outpatient department of obstetrics of two major hospitals in Wuhan, Mainland China from April 2021 to July 2021. Wuhan is the largest city in China in terms of the urban area, and based on the Hubei Statistical Yearbook, Wuhan’s birth rate was 8.97% in 2021. Namely, there are 8.97 births for every 1000 people per year. The two hospitals were comprehensive tertiary hospitals (Zhongnan Hospital affiliated with Wuhan University, and Renmin Hospital of Wuhan University), which are the largest providers of maternity services in Hubei Province, and they provide healthcare services to Chinese-speaking patients from various places.

In this study, women were recruited from the outpatient department of obstetrics by two researchers who distributed flyers containing an outline of the study content. Researchers provided an in-person, verbal explanation, introduction of the study’s aim, and research significance before participants filled out the questionnaire. Research flyers were also posted with a quick response code, so that the women could scan it and jump to the link for informed consent. After they completed the informed consent, the women were required to answer the online questionnaires. We recruited women in the third trimester, because research argued that FOC may increase as the pregnancy progresses, and it was highest in the third trimester [25]. Eligible women had to be (1) pregnant; (2) in the third trimester; (3) over 18 years old; and (4) willing to participate in this study. Women who had a cognitive impairment or communication barriers in oral or written Chinese were excluded. There was no compensation or monetary benefit for their participation. The Research Ethics Board from the medical school at Wuhan University approved this study (2019YF2019). All methods were carried out in line with relevant guidelines and regulations of the Committee of Zhongnan Hospital, Renmin Hospital, and the medical school at Wuhan University.

### Sample size

According to the requirements for factor analysis, the sample size should be at least 5 participants for each item [26]. The W-DEQ-A comprises 33 items, so the intended sample size was estimated to be greater than 165. Given the 20% invalid questionnaires, a sample size of at least 207 women was considered the minimum required. Based on recommendations of previous literature, the appropriate sample sizes for exploratory factor analysis (EFA) and confirmatory factor analysis (CFA) should both be larger than 150, and the recommended sample size is at least 300 [27, 28]. Initially, a total of 378 questionnaires were distributed. Among them, 14 invalid questionnaires were eliminated because more than 5% of the items were missing. A total of 364 participants were analyzed in the study.

### Instruments

The questionnaire measured the general characteristics of pregnant women, including age, height, gestational weeks, weight at present, weight before gestation, marital status, education level, resident place, and average monthly family income.

The W-DEQ-A is a 33-item self-report questionnaire, with a 6-point Likert scale ranging from 0 (“not at all”) to 5 (“extremely”). These items can estimate the level of fear regarding childbirth before parturition. Positively formulated questions (item numbers 2, 3, 6, 7, 8, 11, 12, 15, 19, 20, 24, 25, 27, 31) are reversed for calculating sum score. The range of the sum score is from 0 to 165. Besides, a score of 85 or higher indicates clinical fear. A higher total score indicates a more serious fear of childbirth. Original authors of the scale explored psychometric evaluation in 1998 and found an overall reliability of 0.93 [1].

Trait anxiety is a common psychological problem during pregnancy. Fear and anxiety may occur together or independently. Therefore, we also investigated anxiety status and set trait anxiety as a calibration. State-Trait Anxiety Inventory—Trait subscale (STAI-T) consists of 20 items and assesses trait anxiety levels across situations and time. It is scored in a 4-point Likert type rating from 1 (“almost never”) to 4 (“almost always”); the higher the sum score, the higher the anxiety levels. The reliability of STAI-T was 0.827 [29].

### Cultural translation procedure

The study procedure involved translation of W-DEQ-A into the Mandarin Chinese language to conduct cultural adaptation before data collection. With the permission of the author, forward translations were performed. Two bilingual specialists separately translated the original scale into Chinese. Then, they reached a consensus by discussion and agreed on one single Chinese version. After that, the other translator was blinded to relevant concepts and retranslated the Chinese version back into English. The back-translation version was reviewed to confirm any inconsistency and accuracy. Two clinical nursing experts and two linguists reviewed translation contents and processes to eliminate any discrepancy of idioms and semantics between the Chinese version and the original version. Thus, the version used in this study was finalized. A pilot trial of 30 pregnant women was conducted face to face by an investigator, the goal being to assess whether the pregnant women could understand the expressions of the scale. Feedback was obtained from these women on understanding and potential problems. These samples were not a part of the final data collection for this study.

### Statistical analysis

Descriptive statistics, such as Mean and Standard deviation, were used to describe sociodemographic variables. To validate the W-DEQ-A in the Chinese population, the content validity, structural validity, criterion-related validity, convergent validity and reliability of the scale were tested.

Content validity had appraised by the expert panel, which consisted of two associate professors of nursing and three clinical nursing experts in obstetrics and gynecology. Experts rated all items to evaluate the necessity by using a 4-point rating scale: (1) very relevant, (2) relatively relevant with minimal modification, (3) must be changed or not relevant, and (4) not relevant at all. Content validity indices (CVI) > 0.8 or more was considered to be a psychometric satisfaction level of content validity [30].

The structural validity was tested through EFA and CFA, which was used to confirm the latent structure of the instrument. The data were randomly divided into two groups which were used to test EFA (*n* = 182) and CFA (*n* = 182), respectively. The use of Kaiser-Meyer-Olkin (KMO) and Bartlett’s test of sphericity was to check the suitability of the data for EFA. The Maximum Likelihood analysis with maximum variance orthogonal rotation was used on analysis. KMO measure recommended value was 0.6 [31]. The loading of the item ≥ 0.4 was assigned to a factor [32]. To recognize the number of factors, initial eigenvalues were over Kaiser’s Rule of 1 [33], and we also observed the Scree plot. CFA was conducted to assess the model fitness by fit indices, including Chi-square and degrees of freedom ratio (χ ^2^/df), Incremental Fit Index (IFI), Tucker Lewis Index (TLI), Comparative Fit Index (CFI) and Root Mean Square Error of Approximation (RMSEA). When χ ^2^/df was less than 3.0 [34], RMSEA was close to 0.1, and IFI, CFI and TLI were at least 0.7 or higher [35, 36], the model was considered tolerable and confirmable. The criterion-related validity was tested by using Pearson’s correlation, and it examined whether the test score on the scale was correlated with the criterion on an existing scale measured at the same time. In the study, we selected a relevant variable and the corresponding scale based on the previous literature and the experience of researchers, namely the STAI-T [29]. When the correlation coefficient value ranged from 0.4 to 0.8, it was regarded as the secured criterion-related validity [37].

Convergent validity indicated the level of correlation of multiple items of the same factor that were in agreement. We calculated the average variance extracted (AVE) and composite reliability (CR). The AVE value should be close to 0.5 or higher [38], and the CR value should be well-above the benchmark of 0.7 [39]. On the other hand, the content consistency reliability was evaluated with Cronbach’s alpha. Generally, it was considered an acceptable value when Cronbach’s alpha coefficient was greater than 0.7 [40]. Reverse indicators were directionally aligned to ensure the direction consistency. The data collection was analyzed using R studio 4.0.3 [41].

## Results

### Characteristics of the participants and descriptions of W-DEQ-A

A total of 364 pregnant women was recruited. The mean age of the participants was 29.7 ± 3.8 years, while ranging from 20 to 45 years. The average gestational weeks was 34.5 ± 3.3 weeks. There were 8 women who were single. In terms of education level, 3% of them had high school level or below, and the high monthly income level (≥ 10,000) accounted for 28.3% of the total. Table 1 shows the participants’ characteristics. The average score of W-DEQ-A was 41.41 ± 7.92. The lowest scored item was item 5 “Confident” with mean ± SD of 1.63 ± 0.80. Item 4 “Strong” had the highest scores with mean ± SD of 2.49 ± 0.94. Sixty-three (17.3%) participants had clinical fear with a score ≥ 85.

**Table 1:** Participants’ characteristics

Characteristics	Descriptive statistics	Participant (*N* = 364)
Age (years)	N (Missing)	364 (0)
	Mean (SD)	29.73 (3.83)
	Median (Q1, Q3)	30 (27, 32)
	Range	20–45
Height (cm)	N (Missing)	364 (0)
	Mean (SD)	162.82 (4.85)
	Median (Q1, Q3)	163 (160, 167)
	Range	144–174
Gestational weeks (weeks)	N (Missing)	364 (0)
	Mean (SD)	34.51 (3.30)
	Median (Q1, Q3)	35 (32, 37)
	Range	28–41
Weight at present (kg)	N (Missing)	364 (0)
	Mean (SD)	65.93 (9.49)
	Median (Q1, Q3)	66 (60, 71)
	Range	40–115
Weight before gestation (kg)	N (Missing)	364 (0)
	Mean (SD)	55.50 (7.98)
	Median (Q1, Q3)	55 (50, 60)
	Range	37.5–90
Marital status	N (Missing)	364 (0)
	Unmarried, N (%)	7 (1.9)
	Marital, N (%)	356 (97.8)
	Divorce, N (%)	1 ( 0.3)
Educational level (%)	N (Missing)	364 (0)
	High school or below, N (%)	11 ( 3.0)
	College degree, N (%)	55 (15.1)
	Bachelor degree, N (%)	251 (69.0)
	Master degree or above, N (%)	47 (12.9)
Resident place (%)	N (Missing)	364 (0)
	City, N (%)	303 (83.2)
	Town, N (%)	37 (10.2)
	Countryside, N (%)	24 (6.6)
Average monthly family income (RMB)	N (Missing)	364 (0)
	≤ 2999, N (%)	10 (2.7)
	3000–4999, N (%)	77 (21.2)
	5000–9999, N (%)	174 (47.8)
	≥ 10,000, N (%)	103 (28.3)

*SD* Standard deviations; The number of non-missing persons in each group was used as the denominator to calculate the percentage

### Validity and reliability

The expert panel assessed each item under Chinese culture, especially the relevance, clarity, equivalence of semantics, and cultural suitability. A minor modification was made to item 32 based on experts’ suggestions, namely “Child will die” was changed to “Child departed”. CVI in our results was 0.99 greater than 0.8 for all items, and all 33 items were retained.

**Fig. 1:**
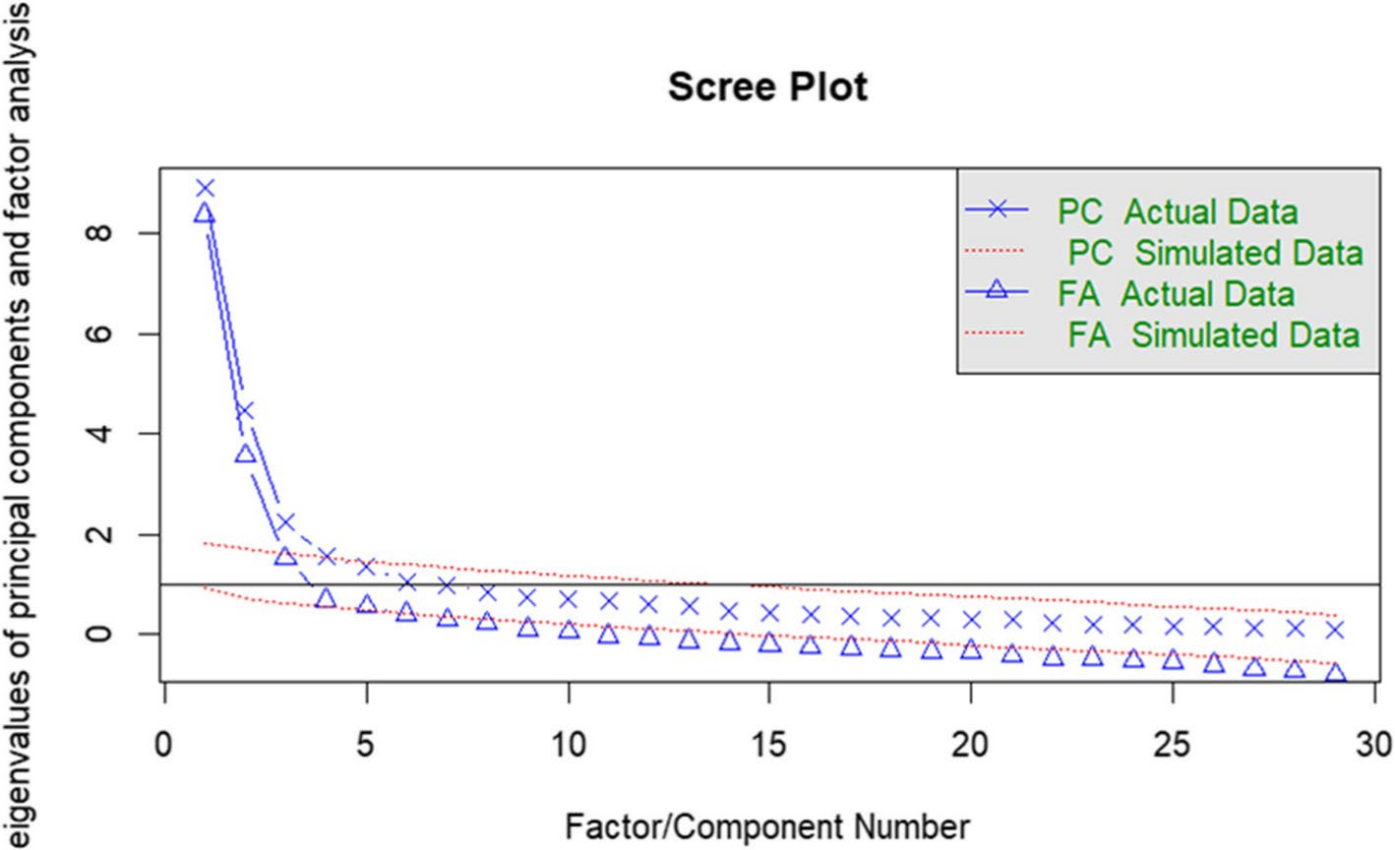
Scree plot and eigenvalue of exploratory factor analysis

Participants in our study were randomly halved by using R’s psych package, where 182 samples were subjected to EFA and the other 182 samples were processed for CFA. When EFA was conducted, factorability was performed with Bartlett’s test of sphericity and KMO test to determine the sample size adequacy and the samples’ fit. The KMO value was 0.85 greater than the recommended value of 0.6 [31], with 528 degrees of freedom, 7.007 of χ^2^/df values and the Chi-square statistic was 3699.953. Bartlett’s measure was confirmed, and the significance level was < 0.001, showing the variables were suitable for the factor analysis. We tested the first EFA on 33 items, and then extracted 5 factors through Maximum Likelihood analysis with maximum variance orthogonal rotation, which could explain 54% of the total variance. The 4 items with a minimum factor loading of 0.4 were excluded. Analyzing the second EFA using the rest of items with 29, the scree plot showed 5 common factors (Fig. 1), explaining 57% of the total variance. No items were with low factor loadings at this time (Table 2). Each factor solution showed a high eigenvalue and all items had substantial loadings into a single factor. Factor 1 (“Lack of self-efficacy”) included 16 items; factor 2 (“Negative appraisal”), factor 3 (“Isolation”), factor 4 (“Concern for the child”), and factor 5 (“Fear”) had 4, 3, 2, and 4 items, respectively.

**Table 2:** Factorial load matrix of exploratory factor analysis (*n* = 182)

Items	Factor 1	Factor 2	Factor 3	Factor 4	Factor 5
1. Fantastic	0.56				
4. Strong	0.61				
5. Confident	0.81				
9. Safe	0.66				
10.Independent	0.73				
13. Glad	0.80				
14. Proud	0.84				
16. Composed	0.85				
17. Relaxed	0.87				
18. Happy	0.54				
21. Longing for child	0.52				
22. Self-confidence	0.70				
23. Trust	0.59				
28. Funny	0.44				
29. Natural	0.56				
30. Obvious	0.47				
19. Panic		0.58			
24. Pain		0.44			
25. Behave badly		0.79			
27. Lose control		0.73			
11. Desolate			0.73		
15. Abandoned			0.76		
20. Hopelessness			0.64		
32. Child will die				0.94	
33. Child will be injured				0.93	
6. Afraid					0.55
7. Deserted					0.61
8. Weak					0.55
12. Tense					0.68

Factor 1: Lack of self-efficacy; factor 2: Negative appraisal; factor 3: Isolation; factor 4: Concern for the child; factor 5: Fear

**Fig. 2:**
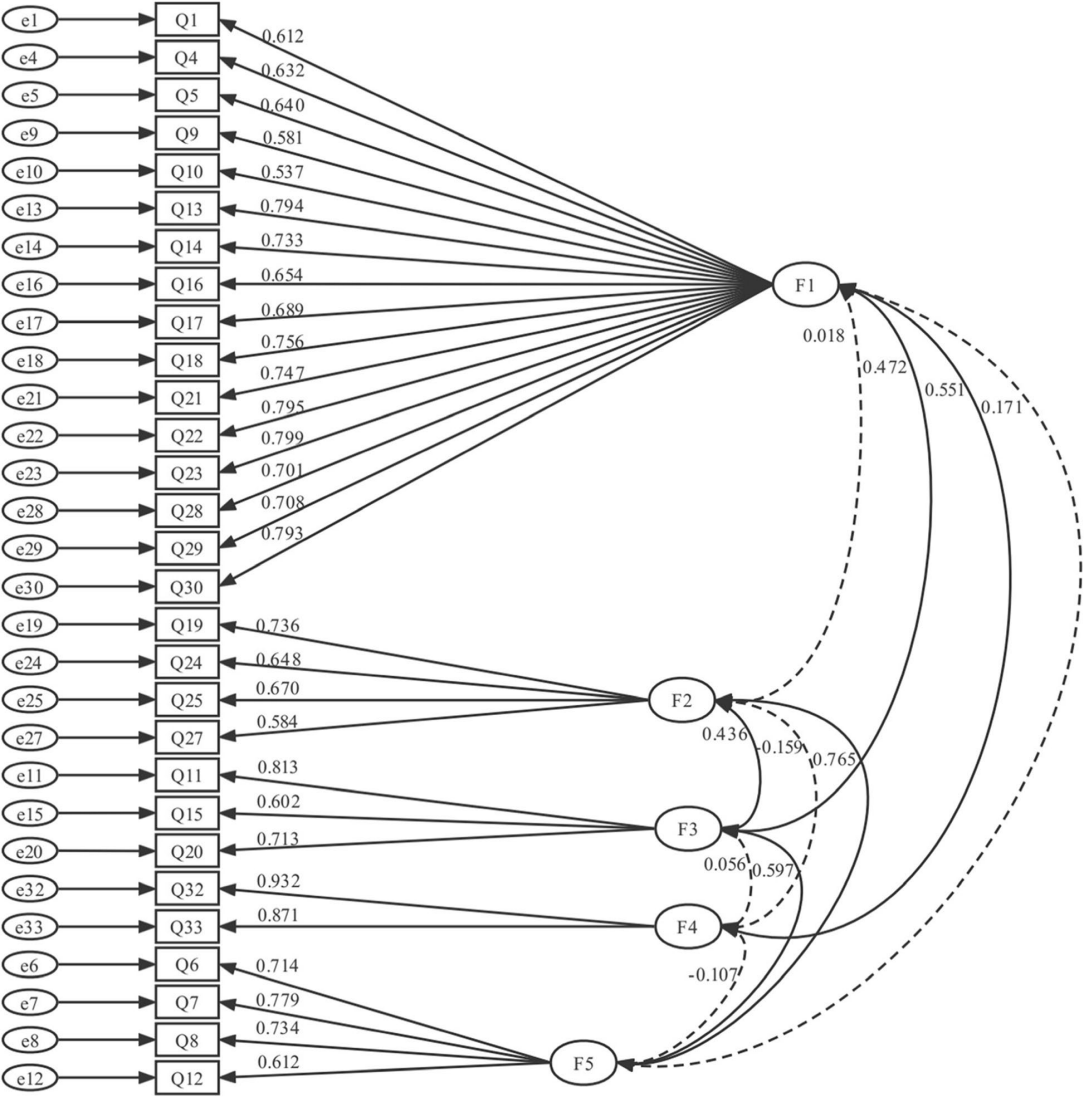
Confirmatory factor analysis standardized item loadings and factor correlations (*n* = 182; *P* < 0.05). Dashed lines represent no statistical significance, solid lines represent statistical significance. Q1-Q33 = questionnaire items 1–33; F1 = factor 1 “Lack of self-efficacy”; F2 = factor 2 “Negative appraisal”; F3 = factor 3 “Isolation”; F4 = factor 4 “Concern for the child”; F5 = factor 5 “Fear”

Structural equation modeling was conducted to analyze the CFA. For the goodness-of-fit indices, CFI was 0.771 (χ ^2^/df = 2.96), and other indices were shown in Table 3. CFA standardized item loadings and factor correlations was illustrated in Fig. 2. As for the criterion-related validity, STAI-T was regarded as a criterion. The correlation between the W-DEQ-A and STAI-T total scores was measured, and the final correlation coefficient was 0.531 (*t* = 11.897; df = 362; *P* < 0.05), revealing a significant and acceptable correlation between them.

**Table 3:** Fit model indices (*n* = 182)

Absolute Fit Indexes	result	Incremental Fit Indexes	result
χ ^2^/df	2.96	IFI	0.774
RMSEA	0.104	TLI	0.747
		CFI	0.771

*IFI* Incremental Fit Index, *TLI* Tucker Lewis Index, *CFI* Comparative Fit Index, *RMSEA* Root Mean Square Error of Approximation

The AVE values were all close to or greater than 0.5, and the CR values of all factors were more than 0.7, conforming the acceptable convergent validity (Table 4). Additionally, Cronbach’s alpha for the total scale and each factor were all above 0.7, especially the total scale of Cronbach’s alpha was up to 0.911, which indicated that it had a good level of internal consistency. Cronbach’s alpha of each construct was presented in Table 4.

**Table 4:** Convergent validity and reliability

	AVE	CR	Cronbach’s alpha (95% CI)
Factor 1	0.496	0.939	0.939 (0.920 ~ 0.952)
Factor 2	0.439	0.756	0.755 (0.674 ~ 0.810)
Factor 3	0.508	0.753	0.751 (0.645 ~ 0.821)
Factor 4	0.813	0.897	0.896 (0.841 ~ 0.937)
Factor 5	0.505	0.802	0.738 (0.647 ~ 0.806)
Total			0.911 (0.888 ~ 0.928)

*AVE* Average variance extracted, *CR* Composite reliability; Factor 1: Lack of self-efficacy; factor 2: Negative appraisal; factor 3: Isolation; factor 4: Concern for the child; factor 5: Fear

## Discussion

Evidence on screening tools which assess for FOC is crucial for Chinese nursing personnel to strengthen interventions. However, there are few studies on FOC in China and a lack of reliable and valid instruments to analyze this issue. This study is the first research trial that explored the psychometric evaluation of the Mandarin Chinese version of W-DEQ-A in a large sample. It indicated that the scale is reliable and valid to measure FOC in pregnant women. Therefore, this study provides guidance for assessing the levels of fear in women prior to delivery, and promotes the application of this scale.

We found that 17.3% of pregnant women experience clinical fear, compared to the 11% of women in European countries as found in the previous study [12]. This highlights the need to focus on the mental health of pregnant women. The proportion of people with sum scores greater than 85 was more than the proportion from a Norwegian study [42]. It is speculated that FOC is influenced by many factors, and is temporal and geographical, so the level of fear may vary among women in different countries. The highest scores were from item 4 “Strong”, and participants did not think they were strong enough. A possible reason is that the labor is an uncontrollable process, and women may worry that they will not do well. This suggests that nurses and midwives need to give more psychological care.

Due to psychometrics difference in different cultures, we need to test the scale to translation and cultural adaptation applied in East Asian countries through a sequential standardization and well-documented process [43]. We had a study team of professional members, the support of the original author, and expert panel conduct a full review to ensure the quality of the scale. Furthermore, there was no systematic withdrawal of participants during the survey, which made the sample representative. Because we recruited participates face-to-face, there may be less response bias than with recruitment from network. According to suggestions of our experts, we modified the expression of the item 32, “Child will die”. In Chinese culture, the death of the child is a taboo matter for a family, so we revised it to a more euphemistic expression about death. Then, items were deemed understandable and acceptable, revealing acceptable content validity.

We found that KMO values were 0.9, indicating sample size adequacy, and justified that the scale fitted factor analysis for items at the significance level using Bartlett’s test of sphericity. The 5 factors identified using EFA were different from the factor structure of other language versions of W-DEQ-A, for example, the Persian version extracted six factors [22, 23], and a four-factor structure was explored relatively in the Japanese version [44]. Interestingly, in the original validation study, it supported a unidimensional model [1], while this study and other language version studies extracted multiple factors and failed to support this view. We thought different dimensions may be due to cultural differences or insufficient data. Moreover, the items of the factor “Concern for the child” was consistent with the Slovak version [45]. In addition, based on the standard of model fitting indices, χ ^2^/df was 2.96 less than 3.0, RMSEA was 0.104 approximate to 0.1, and IFI, TLI, and CFI were greater than 0.7. The model fitting indices obtained by CFA showed that the model fitted was suitable.

Results revealed that the criterion-related validity of W-DEQ-A was satisfactory, and the W-DEQ-A had a great correlation with the STAI-T, which was consistent with Wijma and colleagues’ findings [1]. During late pregnancy, women’s hormone levels change, and they may become both sensitive and vulnerable, making them susceptible to fear and anxiety. When they have expectations about fear, it can impact their anxiety, in turn, increasing their fear and creating a vicious cycle. What’s more, all values of AVE and CR met the acceptable criterion, so convergent validity was confirmed. Then the Cronbach’s alpha coefficient for the total scale in our study was 0.911 greater than 0.7 [40], showing sufficient internal consistency and excellent reliability. The Cronbach’s alpha is also similar to the results of the original scale [1], and is higher than an Iranian study [23].

Many efforts have been made to ensure the credibility of the study, such as reducing information bias, decreasing random errors, rigorous study design, and improving response rates. However, there were still several limitations. First, all participants were in the third trimester, so the results were not representative of the general population of pregnant women, especially in other pregnancy trimesters. Therefore, these samples influenced the generalizability of the findings. We can conduct a longitudinal examination of FOC in the future and add information about fear situations in other pregnancy trimesters. Second, the study lacked some general fear scales to perform a correlated validity. Lastly, this study and other language version studies (such as the Persian version and Japanese version) extracted multiple factors and failed to support a unidimensional model as the original questionnaire, and it may be the cause of the position setting. Future studies should expand sample size (such as a multicenter sample) and consider continuing to validate the model.

## Conclusion

The Chinese version of W-DEQ-A had good reliability and validity to evaluate FOC for pregnant women speaking Mandarin Chinese. Assessment of FOC could help monitor women’s birth experience. It is recommended to pay a close attention to pregnant women’s fears and negative attitudes towards childbirth so that effective interventions can be provided in clinical care settings.

## Data Availability

The datasets used and/or analyzed during the current study are available from the corresponding author on reasonable request.

## Abbreviations

FOC: Fear of childbirth
W-DEQ-A: Wijma Delivery Expectancy/Experience Questionnaire Version A
CAQ: Childbirth Attitude Questionnaire
VAS: Visual Analogue Scale
FOBS: Fear of Birth Scale
W-DEQ: Wijma Delivery Expectancy/Experience Questionnaire
STAI-T: State-Trait Anxiety Inventory—Trait subscale
EFA: Exploratory factor analysis
CFA: Confirmatory factor analysis
KMO: Kaiser-Meyer-Olkin
CVI: Content validity indices
IFI: Incremental Fit Index
TLI: Tucker Lewis Index
CFI: Comparative Fit Index
RMSEA: Root Mean Square Error of Approximation
AVE: Average variance extracted
CR: Composite reliability
